# Real-World Outcomes of Lenvatinib in Radioactive Iodine-Refractory Differentiated Thyroid Cancer: A Multicentre Retrospective Study

**DOI:** 10.3390/cancers18172881

**Published:** 2026-09-06

**Authors:** Wing-Lok Chan, Winnie Wing-Yan Tin, Kenneth Chun-Wai Wong, Carol Chi-Hei Kwok, James C. H. Chow, Tsz-Chim Liu, Gavin Tin-Chun Cheung, Wesley Yuen-Lum Choi, Lok-Sze Joyce Au, Jenny Ching-Hei To, Grace Yuk-Fong Lo, Li-Yu Hou, Kary King-Tung Yeung, Hoi-Leung Leung, Dora Lai-Wan Kwong

**Affiliations:** 1 Department of Clinical Oncology, School of Clinical Medicine, Li Ka Shing Faculty of Medicine, The University of Hong Kong, Hong Kong SAR, China; 2 Department of Clinical Oncology, Tuen Mun Hospital, Hong Kong SAR, China; 3 Department of Clinical Oncology, Prince of Wales Hospital, Hong Kong SAR, China; 4 Department of Oncology, Princess Margaret Hospital, Hong Kong SAR, China; 5 Department of Clinical Oncology, Queen Elizabeth Hospital, Hong Kong SAR, China; 6 Department of Clinical Oncology, Pamela Youde Nethersole Eastern Hospital, Hong Kong SAR, China; 7 Department of Oncology, United Christian Hospital, Hong Kong SAR, China; 8 Department of Clinical Oncology, Queen Mary Hospital, Hong Kong SAR, China

**Keywords:** lenatinib, radioactive iodine refractory, differentiated thyroid cancer, dose modification, progression-free survival

## Abstract

**Background:** Lenvatinib is an established first-line therapy for radioactive iodine (RAI)-refractory differentiated thyroid cancer (DTC); however, real-world data, particularly from Asian populations with long-term follow-up, remain limited. **Methods:** We conducted a multicentre retrospective cohort study across seven public hospitals in Hong Kong. Adult patients with RAI-refractory DTC who received lenvatinib between 1 January 2010 and 31 March 2025 were included. Efficacy outcomes included progression-free survival (PFS), overall survival (OS), objective response rate (ORR), and duration of treatment. Landmark analyses were performed at 6 and 12 months after lenvatinib initiation. Safety outcomes included treatment-related adverse events graded according to CTCAE version 5.0. **Results:** A total of 82 patients were included, with a median follow-up of 24.7 (IQR, 14.2–46.6) months. The median age at treatment initiation was 69 years, and the median starting dose of lenvatinib was 14 mg. Median PFS was 31.0 months (95% CI, 16.6–40.8) and median OS was 46.8 months (95% CI, 33.6–67.6), with multivariable analyses identifying brain metastasis as the most consistent adverse prognostic factor. The ORR was 52.4% and the DCR was 82.9%. Treatment-related adverse events were common, most frequently hypertension (73.2%) and proteinuria (57.3%); grade ≥ 3 events occurred predominantly as hypertension (45.1%). **Conclusions:** Lenvatinib achieved durable clinical outcomes in patients with RAI-refractory differentiated thyroid cancer despite the frequent use of reduced starting doses. Treatment-related toxicities were common but generally manageable, allowing prolonged treatment in many patients.

## Simple Summary

Lenvatinib is an established treatment for radioactive iodine-refractory differentiated thyroid cancer, but long-term real-world data remain limited, particularly in Asian populations. In this multicentre study across seven public hospitals in Hong Kong, we evaluated the effectiveness and safety of lenvatinib in 82 patients. Despite the frequent use of reduced starting doses, lenvatinib demonstrated durable clinical activity with manageable toxicity. Dose reduction and temporary treatment interruption were common and often allowed treatment continuation following adverse events. These findings provide real-world evidence on treatment patterns and outcomes in routine practice and suggest that careful toxicity management may facilitate long-term treatment delivery.

## 1. Introduction

Differentiated thyroid cancer (DTC) accounts for approximately 90% of all thyroid malignancies and is generally associated with a favorable prognosis [1,2]. However, 5–15% of patients develop progressive disease that becomes refractory to radioactive iodine (RAI) therapy, a condition associated with poor outcomes and a reported 10-year survival rate of approximately 10% [3,4,5,6].

Lenvatinib, an oral multitargeted tyrosine kinase inhibitor, demonstrated significant improvements in progression-free survival (PFS) and tumor response compared with placebo in the pivotal phase III SELECT trial, and has since become the standard first-line systemic therapy for patients with RAI-refractory DTC [7]. However, patients enrolled in the SELECT study were highly selected, with most having an Eastern Cooperative Oncology Group (ECOG) performance status of 0–1, a median age in the early 60s, and a predominantly non-Asian population (80%). As a result, trial findings may not fully reflect outcomes in routine clinical practice, where patients are often older, have a greater burden of comorbidities, and require more individualized treatment strategies, including modified starting doses and early dose adjustments.

Although multiple real-world studies have evaluated lenvatinib in RR-DTC, many were conducted in single institutions, included limited patient numbers, or had relatively short follow-up [8,9,10,11,12,13,14,15,16,17,18]. Furthermore, data from territory-wide healthcare systems and contemporary Asian multicentre cohorts with extended follow-up remain limited. Additional evidence is therefore needed to better characterize long-term effectiveness, toxicity patterns, and treatment modifications in routine clinical practice.

We therefore conducted a multicentre retrospective cohort study to evaluate the real-world efficacy and safety of lenvatinib in patients with RAI-refractory differentiated thyroid cancer across seven public hospitals in Hong Kong, with a focus on long-term outcomes, treatment patterns, and treatment-related toxicities.

## 2. Materials and Methods

### 2.1. Study Aim

This study aimed to evaluate the real-world efficacy and safety of lenvatinib in patients with RAI-refractory DTC, and to characterize treatment patterns and factors associated with clinical outcomes in routine clinical practice.

### 2.2. Study Design and Setting

This was a multicentre retrospective cohort study conducted across seven hospitals under the Hospital Authority in Hong Kong, including Queen Mary Hospital, Tuen Mun Hospital, Queen Elizabeth Hospital, United Christian Hospital, Princess Margaret Hospital, Prince of Wales Hospital, and Pamela Youde Nethersole Eastern Hospital. The study was conducted in accordance with the Declaration of Helsinki and approved by the Hospital Authority Central Institutional Review Board (IRB Reference Number: CIRB-2025-673-1). Informed consent was waived owing to the retrospective nature of the study and the use of anonymized data.

### 2.3. Patient Eligibility

Eligible patients were adults (≥18 years) with histologically confirmed differentiated thyroid carcinoma who received lenvatinib between 1 January 2010 and 31 March 2025.

RAI-refractory (RAI-R) disease was defined by one or more of the following criteria: (1) metastatic lesions without radioactive iodine uptake; (2) loss of previously demonstrated radioactive iodine avidity; (3) heterogeneous uptake, with some lesions demonstrating uptake while others did not; or (4) progression despite radioactive iodine uptake. Patients were eligible for lenvatinib if they had RAI-R disease with clinically significant progression and/or symptoms requiring systemic therapy.

As this was a retrospective multicentre study, no central review of radioactive iodine scans or treatment eligibility was performed. Consecutive eligible patients who received lenvatinib during the study period were included.

### 2.4. Data Collection and Treatment Exposure

Clinical data were extracted from the Hospital Authority Clinical Management System using standardized data collection templates across participating centres. Patients were followed from initiation of lenvatinib until death or last clinical contact.

Collected variables included demographic characteristics (age and sex), comorbidities, tumour histology, date of initial diagnosis, sites of metastases, and RAI treatment history, including the number of administrations and cumulative dose.

Treatment-related data included lenvatinib starting dose, dose reductions, treatment interruptions, maintenance dose, treatment duration, and treatment discontinuation. Maintenance dose was defined as the last documented lenvatinib dose before permanent discontinuation or last follow-up. Treatment interruption was defined as temporary cessation of lenvatinib followed by treatment resumption.

Radiological response assessments and treatment-related adverse events were recorded. Imaging assessments were generally performed every 3–6 months according to disease status and clinician discretion. The primary reason for treatment discontinuation (e.g., disease progression, treatment-related toxicity, or other clinical factors) was documented. Patients who remained alive at the data cutoff date were censored at their last follow-up.

### 2.5. Outcome Measures

#### 2.5.1. Efficacy Outcomes Measures

Efficacy outcomes included progression-free survival (PFS), overall survival (OS), objective response rate (ORR), disease control rate (DCR), and duration of treatment. PFS was defined as the time from lenvatinib initiation to radiological or clinical progression, or death from any cause. OS was defined as the time from treatment initiation to death from any cause.

Tumour response was assessed using radiological evaluations according to the Response Evaluation Criteria in Solid Tumors (RECIST) version 1.1 [19]. ORR was defined as the proportion of patients achieving a complete or partial response as their best documented response. DCR was defined as the proportion achieving complete response, partial response, or stable disease. Duration of treatment was defined as the time from lenvatinib initiation to permanent discontinuation or death.

#### 2.5.2. Safety Outcomes Measures

Safety outcomes included the incidence, type, and severity of treatment-related adverse events, graded according to the Common Terminology Criteria for Adverse Events (CTCAE) version 5.0. Additional safety outcomes included time to onset of adverse events, treatment interruptions, dose reductions, and treatment discontinuation due to toxicity.

### 2.6. Statistical Analysis

Baseline characteristics were summarized descriptively. Continuous variables were reported as median with range or interquartile range (IQR), as appropriate, while categorical variables were reported as frequencies and percentages.

Survival outcomes were estimated using the Kaplan–Meier method. Patients without an event at the time of analysis were censored at their last follow-up. Multivariable Cox proportional hazards regression was performed to identify factors associated with PFS and OS. Clinically relevant variables were included in the initial multivariable models, and bidirectional stepwise regression was subsequently used to derive the final parsimonious models. The proportional hazards assumption was assessed using Schoenfeld residuals. Hazard ratios (HRs) and 95% confidence intervals (CIs) were reported.

Given the potential for immortal-time bias associated with maintenance dose, landmark analyses were performed at 6 and 12 months after lenvatinib initiation. Patients who remained alive and progression-free at each landmark timepoint were included in the corresponding analysis. Survival times were recalculated from the landmark timepoint. Landmark dose was defined as the lenvatinib dose being received at the corresponding landmark. Separate multivariable Cox regression analyses were performed within each landmark cohort to evaluate associations between starting dose, landmark dose, and survival outcomes.

A two-sided *p*-value < 0.05 was considered statistically significant. Analyses were performed using R version 4.5.2.

## 3. Results

### 3.1. Patient Characteristics

A total of 82 patients with RAI-refractory DTC who received lenvatinib were included. The median follow-up duration was 24.7 (IQR 14.2–46.6) months. The median age at lenvatinib initiation was 69 years (range, 30–89), and 52 patients (63.4%) were female.

Papillary thyroid carcinoma was the most common histological subtype (45 patients, 54.9%), followed by follicular thyroid carcinoma (32 patients, 39.0%) and poorly differentiated thyroid carcinoma (5 patients, 6.1%).

The most common metastatic sites were the lung (73 patients, 89.0%) and bone (42 patients, 51.2%). Lymph node metastases were also common, including cervical lymph nodes in 32 patients (39.0%) and mediastinal lymph nodes in 28 patients (34.2%). Baseline demographic and clinical characteristics are summarized in Table 1.

### 3.2. Treatment Exposure

The median starting dose of lenvatinib was 14 mg (range, 4–24 mg), and the median maintenance dose was 10 mg (range, 2–20 mg). The distribution of starting doses and corresponding dose reductions is summarized in Table 2. Dose reductions were more common among patients receiving higher starting doses, occurring in 37 of 41 patients (90.2%) who received a starting dose ≥14 mg, compared with 23 of 41 patients (56.1%) who received a starting dose <14 mg. Overall, 62 patients (75.6%) had a maintenance dose <10 mg (Table 2).

The median duration of treatment was 15.9 months (IQR, 0.9–94.3 months). Treatment interruption occurred in 67 patients (81.7%), with a median interruption duration of 14 days (IQR, 13–28). The median proportion of time on treatment interruption was 2.30% (IQR, 1.43–9.38%) relative to total treatment duration.

At the time of last follow-up, 55 patients (67.1%) had permanently discontinued lenvatinib. The most common reasons for treatment discontinuation were disease progression (25 patients, 30.5%) and treatment-related toxicity (17 patients, 20.7%). Other reasons included intercurrent medical events such as pneumonia, pancreatitis, and accidental death. 17 patients (20.7%) subsequently received further systemic therapy for RAI-refractory thyroid cancer.

### 3.3. Efficacy Outcomes

#### 3.3.1. Progression-Free Survival and Overall Survival

Landmark analyses were performed at 6 and 12 months after initiation of lenvatinib. At the 6-month landmark, 68 patients remained alive and progression-free and were included in the analysis. During subsequent follow-up, 41 progression events and 28 deaths were observed. The median PFS was 31.0 months (95% CI, 18.6–43.3), and the median OS was 49.2 months (95% CI, 34.7-not reached).

**Figure 1:**
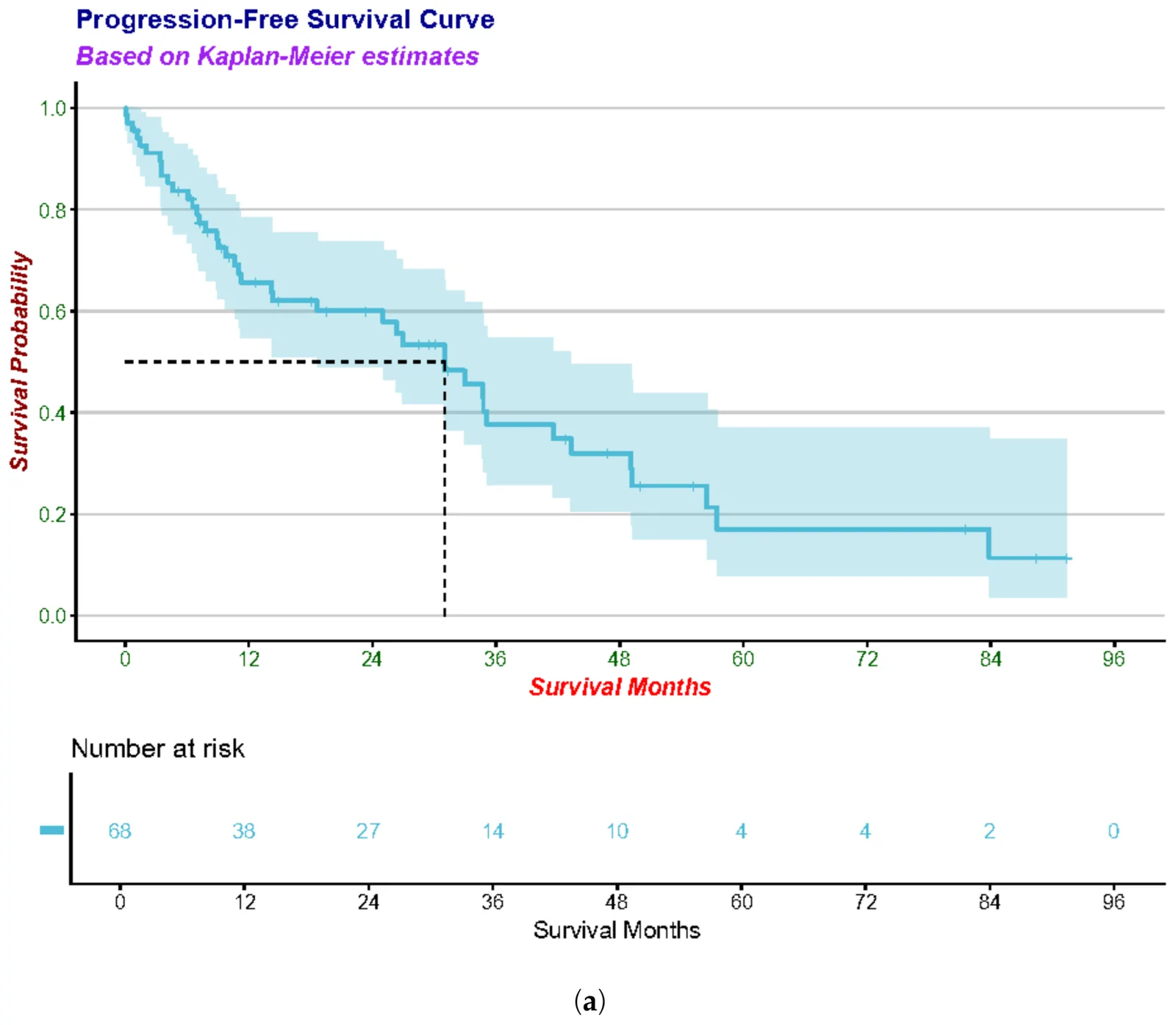

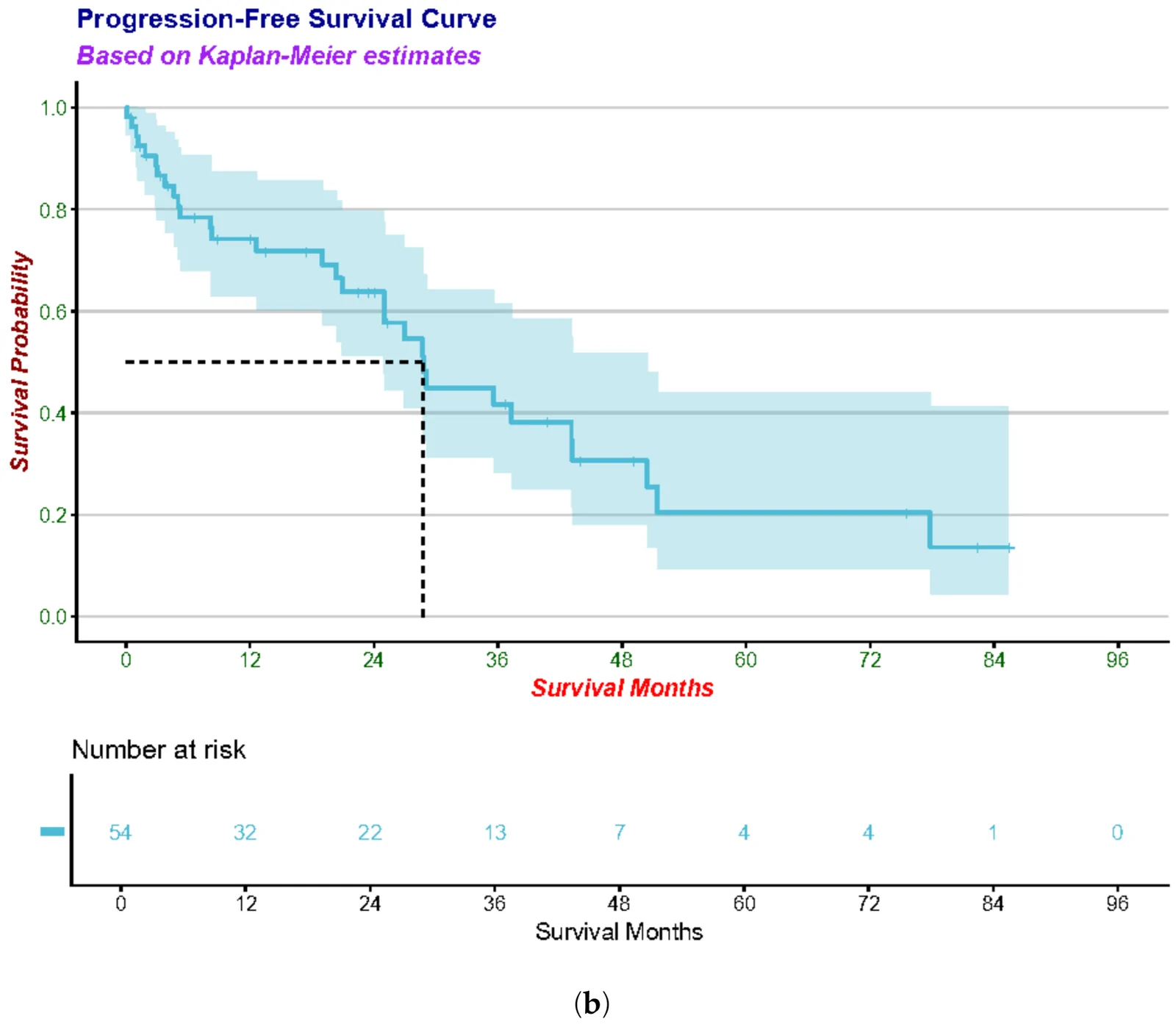
Progression-free survival according to landmark analyses. (**a**) Kaplan–Meier estimates of progression-free survival from the 6-month landmark (n = 68). (**b**) Kaplan–Meier estimates of progression-free survival from the 12-month landmark (n = 54).

**Figure 2:**
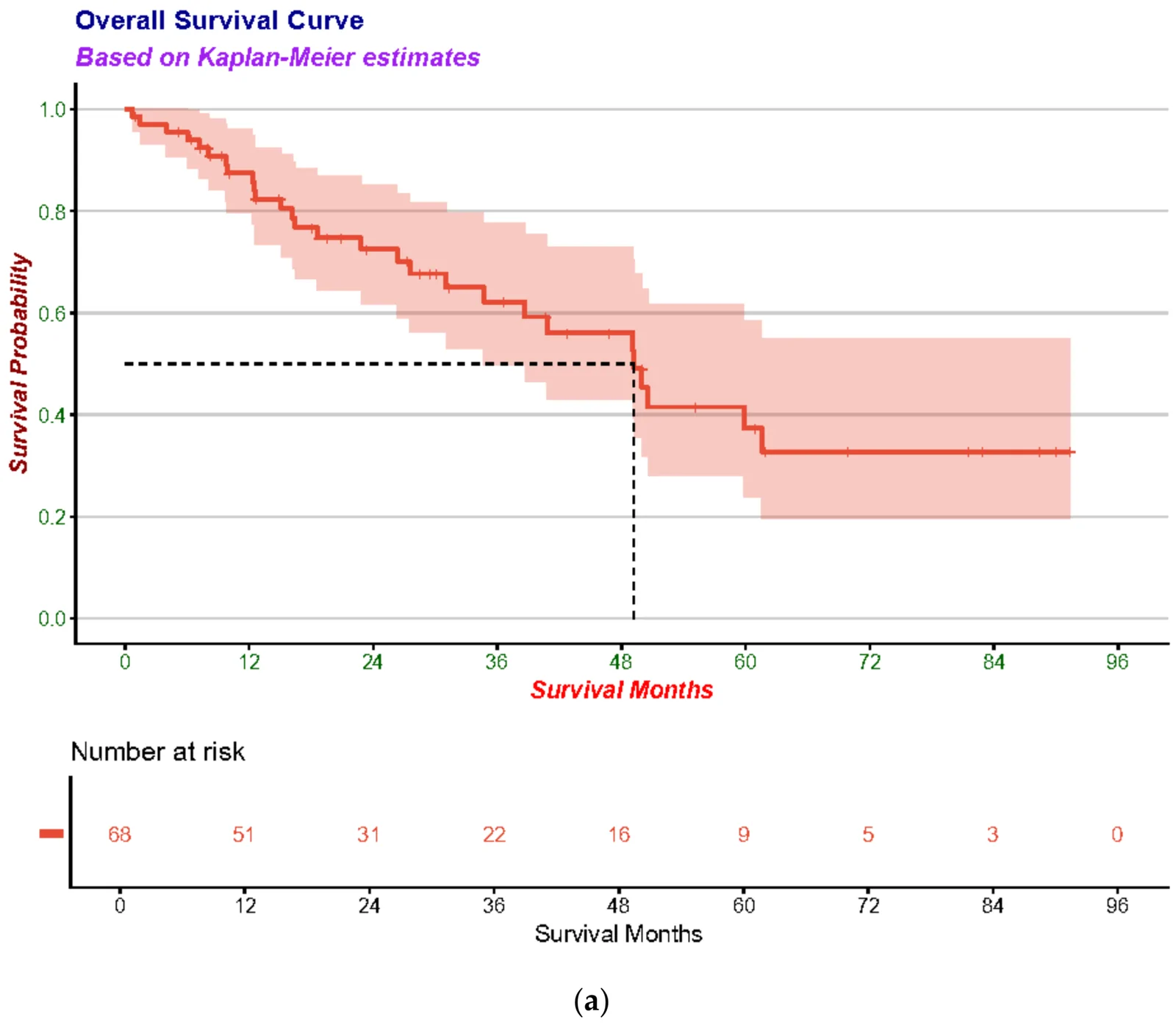

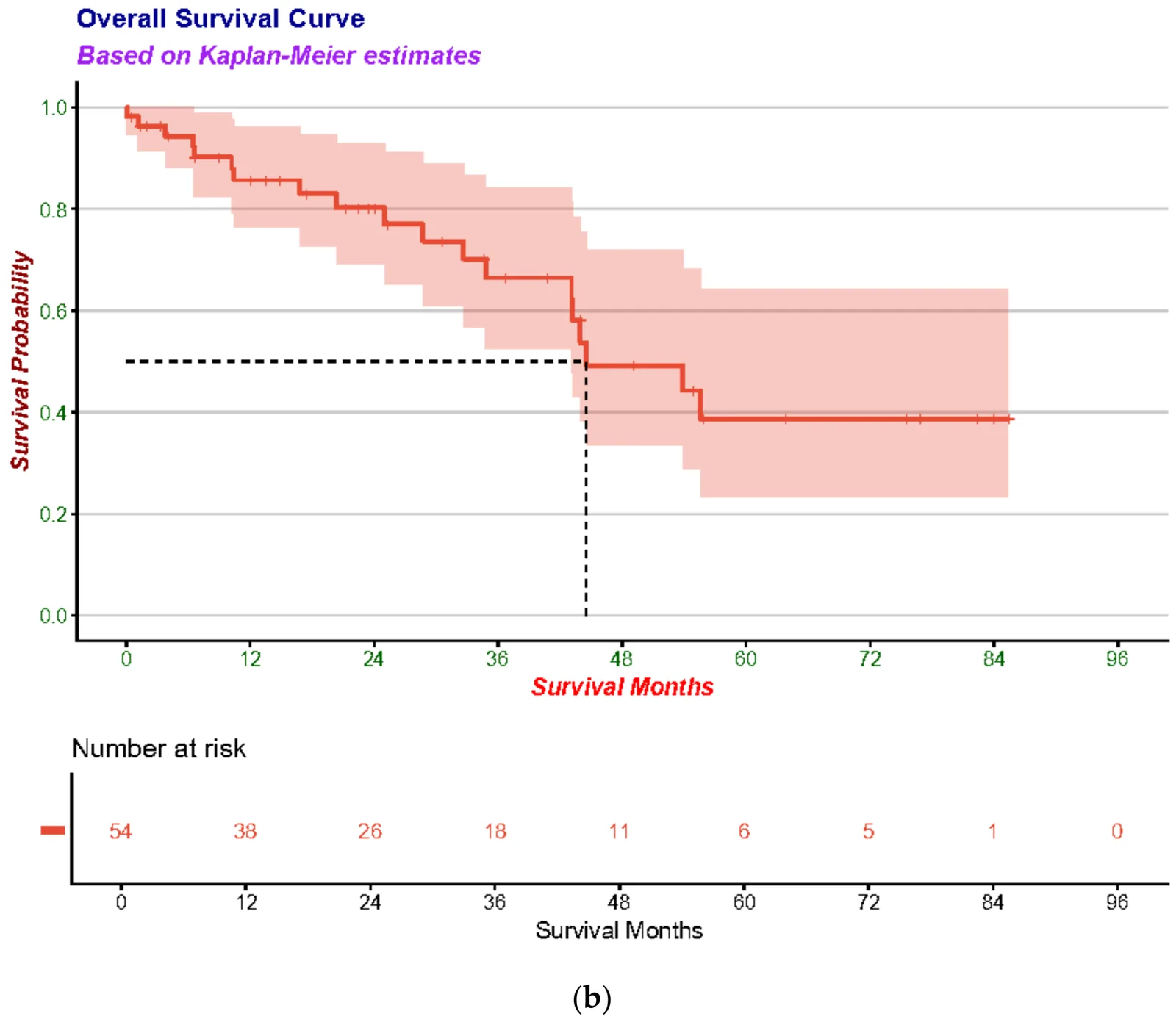
Overall survival according to landmark analyses. (**a**) Kaplan–Meier estimates of overall survival from the 6-month landmark (n = 68). (**b**) Kaplan–Meier estimates of overall survival from the 12-month landmark (n = 54).

At the 12-month landmark, 54 patients remained alive and progression-free and were included in the analysis. During subsequent follow-up, 30 progression events and 19 deaths were observed. The median PFS was 28.8 months (95% CI, 25.0–50.4), and the median OS was 44.5 months (95% CI, 43.1-not reached) (Figure 1 and Figure 2).

Multivariable Cox regression analyses were performed within each landmark cohort (Table 3). In the 6-month landmark analysis, brain metastasis was independently associated with shorter PFS (HR 3.24; 95% CI, 1.21–8.67; *p* = 0.019). This association remained significant in the 12-month landmark analysis (HR 6.33; 95% CI, 1.96–20.46; *p* = 0.02).

For OS, brain metastasis demonstrated a trend towards inferior survival in the 6-month landmark analysis (HR 3.83; 95% CI, 1.2595–11.75; *p* = 0.019) and was independently associated with worse OS in the 12-month landmark analysis (HR 9.21; 95% CI, 2.14–39.60; *p* = 0.003). In addition, a starting dose ≥14 mg was independently associated with improved OS in the 12-month landmark analysis (HR 0.28; 95% CI, 0.08–0.99; *p* = 0.049).

#### 3.3.2. Tumour Response

The ORR was 52.4%, and the DCR was 82.9%. No complete responses were observed. Partial response was achieved in 43 patients (52.4%), stable disease in 25 patients (30.5%), and progressive disease in 9 patients (11.0%). Five patients discontinued treatment prior to formal radiological response assessment and were not evaluable for tumour response.

### 3.4. Safety

Treatment-related adverse events were common. The most frequently observed events were hypertension (60 patients, 73.2%), proteinuria (48 patients, 58.5%), malaise (21 patients, 25.6%), diarrhoea (11 patients, 13.4%), and hand-foot syndrome (11 patients, 13.4%). The median time to onset was 1.74 months for hypertension, 6.76 months for proteinuria, 3.58 months for malaise, 7.30 months for diarrhoea, and 10.47 months for weight loss.

Grade 3–5 adverse events were most commonly hypertension (37 patients, 45.1%) and proteinuria (13 patients, 15.9%). Intracranial haemorrhage occurred in three patients (3.7%), including one grade 3 event (1.2%) and two grade 5 events (2.4%). The times to onset were 1.15, 32.31, and 70.68 months after lenvatinib initiation. None of the affected patients had documented brain metastases or were receiving anticoagulation therapy at the time of the event. One patient developed severe treatment-related hypertension (systolic blood pressure >220 mmHg) before the haemorrhage. In all cases, the diagnosis was confirmed by brain magnetic resonance imaging. Although a causal relationship could not be definitively established, the events were considered possibly related to lenvatinib treatment. Acute kidney injury occurred in two patients (2.4%), including one grade 3 event (1.2%). A detailed summary of treatment-related adverse events is presented in Table 4.

## 4. Discussion

In this multicentre retrospective study across seven public hospitals in Hong Kong, lenvatinib demonstrated durable clinical activity in patients with RAI-refractory differentiated thyroid cancer. Median PFS ranged from 28.8 to 31.0 months, while median OS ranged from 44.5 to 49.2 months. Despite frequent dose reductions and treatment interruptions, clinical outcomes remained favourable and were broadly comparable to those reported in clinical trials and other real-world cohorts. Treatment-related adverse events were common but generally manageable with dose modification and supportive care.

### 4.1. Comparison with Previous Studies

The pivotal phase III SELECT trial established lenvatinib as an effective treatment for RAI-refractory DTC, reporting a median PFS of 18.3 months and substantial improvements in response rate compared with placebo [20]. Although overall survival benefit was more evident in older patients, these patients also experienced greater toxicity and required earlier dose reduction.

Compared with SELECT, patients in our cohort were generally older and more frequently received reduced starting doses. Nevertheless, survival outcomes remained favourable and are broadly consistent with those reported in contemporary real-world studies. A comparison of baseline characteristics, treatment patterns, and clinical outcomes between the present cohort, the SELECT trial, and selected real-world studies is provided in Supplementary Table S1.

Evidence directly comparing reduced and standard starting doses in RAI-refractory DTC remains limited. A randomized trial comparing starting doses of 18 mg and 24 mg demonstrated higher response rates with the higher dose but no significant differences in progression-free or overall survival, and similar findings have been reported in retrospective studies. Reduced starting doses are often selected for older patients or those with significant comorbidities to improve tolerability. In our cohort, the median starting dose was 14 mg. While a starting dose ≥14 mg was associated with improved OS in the 12-month landmark analysis, this finding should be interpreted cautiously given the retrospective design, limited sample size, and potential residual confounding. Overall, the available evidence remains insufficient to define an optimal starting-dose strategy.

### 4.2. Dose Modification, Treatment Interruption, and Treatment Continuity

Dose reduction was common, occurring in 73.2% of patients, and was more frequent among those receiving higher starting doses. Nearly all patients who commenced treatment at doses greater than 14 mg required subsequent dose reduction, including all patients who started at 24 mg. This finding is consistent with the established toxicity profile of lenvatinib.

Treatment interruptions were also frequent, occurring in more than 80% of patients. However, interruptions were generally brief, with a median duration of 14 days and accounting for only a small proportion of the overall treatment course. An exploratory analysis of the SELECT trial demonstrated that lenvatinib remained effective despite treatment interruption, particularly when interruptions accounted for less than 10% of the overall treatment duration [21]. Similarly, temporary interruption was commonly used to manage treatment-related toxicity while allowing treatment to resume once adverse events improved.

Several patients maintained durable disease control despite substantial dose reduction. In some cases, dose reductions to as low as 4 mg daily enabled continued treatment in patients who developed significant toxicities, including grade 3 proteinuria. Many patients were able to continue treatment for prolonged periods following toxicity-related dose adjustment.

### 4.3. Safety

The overall safety profile was consistent with the established toxicity profile of lenvatinib. Hypertension and proteinuria were the most common adverse events and the most frequent grade 3–5 toxicities, often necessitating treatment interruption or dose modification.

Given the occurrence of intracranial haemorrhage, careful blood pressure monitoring and prompt management of treatment-related hypertension remain particularly important. Continued vigilance for rare but potentially serious vascular complications is warranted, especially during prolonged treatment.

### 4.4. Limitations

Several limitations should be acknowledged. The retrospective design introduces potential selection bias and reliance on clinical documentation, which may result in incomplete or inconsistent data capture. The absence of randomization and a comparator group limits causal inference. Treatment strategies, including starting dose, dose modification, and imaging schedules, were not standardized across centres and were subject to clinician discretion. Tumour response assessments were not adjudicated through a formal independent central review process, although radiological responses and treatment-related toxicities were reviewed during retrospective data collection. In addition, key factors such as body weight and relative dose intensity were not systematically evaluated and may have influenced treatment tolerability and outcomes. Finally, the sample size was modest, which may limit statistical power for subgroup analyses and affect generalizability. Nonetheless, the multicentre design and extended follow-up provide valuable insights into the long-term use of lenvatinib in patients with RAI-refractory DTC.

## 5. Conclusions

In this multicentre real-world cohort, lenvatinib achieved durable clinical outcomes in RAI-refractory differentiated thyroid cancer despite the frequent use of reduced starting doses. Treatment-related toxicities were common but generally manageable, allowing prolonged treatment in many patients. These findings provide valuable real-world evidence on the effectiveness and tolerability of lenvatinib in clinical practice.

## Figures and Tables

**Table 1:** Clinical Characteristics of the Participants.

Characteristic		Number	%
Age (years)	Median: 69; Range: 30–89		
Gender	Male	30	36.6%
	Female	52	63.4%
Comorbidities	Hypertension	21	25.6%
	Ischemic heart disease	6	7.3%
	Atrial fibrillation	4	4.9%
	Congestive heart failure	2	2.4%
	Hyperlipidemia	15	18.3%
	Diabetes mellitus	8	9.8%
	Cerebrovascular accident	4	4.9%
	Gout	3	3.7%
	Others	5	6.1%
Histology	Papillary thyroid carcinoma	45	54.9%
	Follicular thyroid carcinoma	32	39.0%
	Poorly differentiated thyroid carcinoma	5	6.1%
Metastatic sites	Lung	73	89.0%
	Bone	42	51.2%
	Cervical lymph node	32	39.0%
	Mediastinal lymph node	28	34.2%
	Brain	10	12.2%
	Liver	7	8.5%
	Pleura	6	7.3%
	Adrenal	2	2.4%
	Renal	2	2.4%
RAI episodes	Median: 2; Range: 0–7		
Cumulative RAI dose	Median: 11 GBq; Range: 3.7–48.1 GBq		

**Table 2:** Starting dose and dose reduction.

Starting Dose (mg)	No. of Patients	%	No. of Patients Needed Dose Reduction	%
4	2	2.4%	0	0%
8	10	12.2%	3	30.0%
10	20	24.4%	12	60.0%
12	9	11.0%	8	88.9%
14	21	25.6%	18	85.7%
16	1	1.2%	1	100.0%
18	1	1.2%	1	100.0%
20	8	9.8%	7	87.5%
24	10	12.2%	10	100.0%

**Table 3:** Multivariable Cox regression analyses for (A) progression-free survival and (B) overall survival in the 6-month and 12-month landmark cohorts.

(**A**) **Progression-Free Survival**							
	**Characteristics**	**6-Month Landmark Analysis**			**12-Month Landmark Analysis**		
		**Hazard Ratio**	**95% CI**	***p*** **Value**	**Hazard Ratio**	**95% CI**	***p*** **Value**
1	Age (continuous)	1.01	0.97–1.06	0.569	1.01	0.95–1.07	0.723
2	Sex: female (male as reference)	1.35	0.69–2.62	0.378	1.38	0.62–3.06	0.431
3	Lung metastasis: Yes (no as reference)	2.17	0.43–11.09	0.351	2.60	0.27–25.11	0.408
4	Liver metastasis: yes (no as reference)	2.69	0.78–9.28	0.118	1.20	0.15–9.62	0.866
5	Brain metastasis: yes (no as reference)	3.24	1.21–8.67	0.019	6.33	1.96–20.46	0.002
6	Starting dose ≥14 mg (<14 mg as reference)	1.23	0.59–2.55	0.581	0.90	0.37–2.17	0.812
7	Landmark dose <10 mg (≥10 mg as reference)	1.37	0.63–2.97	0.424	1.38	0.58–3.30	0.466
(**B**) **Overall Survival**							
	**Characteristics**	**6-Month Landmark Analysis**			**12-Month Landmark Analysis**		
		**Hazard Ratio**	**95% CI**	***p*** **Value**	**Hazard Ratio**	**95% CI**	***p*** **Value**
1	Age (continuous)	1.03	0.98–1.08	0.317	1.02	0.95–1.10	0.563
2	Sex: female (male as reference)	1.75	0.75–4.08	0.192	2.55	0.87–7.41	0.087
3	Lung metastasis: Yes (no as reference)	0.97	0.11–8.69	0.982	0.31	0.03–3.38	0.335
4	Liver metastasis: yes (no as reference)	0.86	0.18–4.01	0.844	0.4	0.04–3.63	0.417
5	Brain metastasis: yes (no as reference)	3.83	1.25–11.75	0.019	9.21	2.14–39.60	0.003
6	Starting dose ≥14 mg (<14 mg as reference)	0.55	0.22–1.35	0.192	0.28	0.08–0.99	0.049
7	Landmark dose <10 mg (≥10 mg as reference)	0.54	0.20–1.40	0.203	0.48	0.15–1.51	0.209

**Table 4:** Treatment-related toxicities.

Side Effects	Total		G1–G2		G3–G5		Median Time to Onset (Months)
	n	%	n	%	n	%	Median (IQR)
1. Hypertension	60	73.8%	23	28.1%	37	45.1%	1.74 (0.81–4.59)
2. Proteinuria	48	58.5%	35	42.7%	13	15.9%	6.76 (2.17–15.96)
3. Malaise	21	25.6%	21	25.6%	-	-	3.58 (0.71–8.84)
4. Diarrhoea	11	13.4%	9	11.0%	2	2.4%	7.30 (2.10–15.75)
5. Hand foot syndrome	11	13.4%	10	12.2%	1	1.2%	1.16 (0.29–1.80)
6. Weight loss	8	9.8%	8	9.8%	-	-	10.47 (5.52–20.48)
7. Thrombocytopenia *	4	4.9%	3	3.7%	1	1.2%	0.92; 1.55; 6.08; 8.28
8. Intracranial haemorrhage *	3	3.7%	-	-	3	3.7%	1.15; 32.31; 70.68
9. Gastrointestinal upset *	2	2.4%	2	2.4%	-	-	1.02; 4.57
10. Acute kidney injury *	2	2.4%	1	1.2%	1	1.2%	0.92; 11.08

* For adverse events occurring in fewer than five patients (n < 5), individual onset times are presented instead of median (IQR). Abbreviation: interquartile range.

## Data Availability

Since the study involves human participants, the data cannot be made freely available in the manuscript, the supplemental files, or a public repository due to ethical restrictions. Data can be requested from the Department of Clinical Oncology, the Centre of Cancer Medicine, LKS Faculty of Medicine, the University of Hong Kong for researchers who meet the criteria for access to confidential data. Interested researchers can send data access requests to winglok@hku.hk.

## Supplementary Materials

The following supporting information can be downloaded at https://www.mdpi.com/article/10.3390/cancers18172881/s1, Table S1: Comparison with SELECT trial and other retrospective studies.

## Abbreviations

The following abbreviations are used in this manuscript:

DTC	Differentiated thyroid cancer
RAI	radioactive iodine
PFS	Progression-free survival
ECOG	Eastern Cooperative Oncology Group
OS	overall survival
ORR	objective response rate
CTCAE	Common Terminology Criteria for Adverse Events
