# Supplementary material for: Real-World Outcomes of Lenvatinib in Radioactive Iodine-Refractory Differentiated Thyroid Cancer: A Multicentre Retrospective Study
Source: Cancers (Basel). 2026 Sep 6;18(17):2881. doi: 10.3390/cancers18172881 (PMC13564596; doi:10.3390/cancers18172881)
Supplement: Supplementary file 1 [file cancers-18-02881-s001.zip › cancers-4500241-supplementary.pdf]

Supplementary Table S1: Comparison with SELECT trial and other retrospective studies

Study	Country	No. of patients	Median age	Median follow-up time	Starting dose	% need dose reduction	Maintenance dose	Overall Survival	Median PFS	Remarks
SELECT trial		261	64		24mg	70%	17mg		18.3 months	
Current cohort	Hong Kong SAR	82	69	30.7 months	Median 14mg	73.2%	Median 10mg	Median: 46.8 months	31.0 months	
Worden et al.[8]	US	308	60	18.9 months (IQR: 12.3-24.7)	24mg (62%)<br>14-20mg (38%)	8%	n/a	Not reached (3-year OS: 57.0%, 95% CI 36.8-72.8)	49.0 months	
Kim et al[9]	South Korea	71	61.7		20mg	34.8%	20mg	--	--	
Onaga et al[10]	Japan	91	70	2.9 years	24mg	n/a	n/a	5.1 years	2.4 years	
Jiang et al.[11]	Taiwan	65	65	17.1 months	10mg (range 4-24 mg)	44.6%	10mg	Not reached (24.1-NR)	26.1 months	
Jerkovich et al.[12]	Argentina	22	60	n/a	24mg	50%	16.1mg	Not reached	13.7 months	
Ruicci et al.[13]	Canada	77	62.9	n/a	10mg (n=21)<br>14mg (n=18)<br>24mg (n=38)	n/a	n/a	n/a	n/a	
Majumder et al.[14]	Australia	64	67	n/a	24mg	23 patients out of 45 needed dose reduction if started on 24mg	n/a	n/a	n/a	
Locati et al.[15]	Italy	96	60	n/a	24mg (n=64)<br>20mg (n=10)<br>14mg (n=11)<br>10mg (n=5)	64.5%	19.2mg	24.2 months	11.9 months	
Peelay et al.[16]	India	27	56	19.7 months	24mg (n=10)<br>20mg (n=4)<br>18mg (n=8)<br>14mg (n=3)<br>10mg (n=2)	55.5%	24mg (n=4)<br>20mg (n=5)<br>18mg (n=4)<br>14mg (n=7)<br>10mg (n=7)	35.3 months	12.2 months	No impact of starting dose on PFS and OS
Rendl et al.[17]	Austria	43	70	n/a	24mg	91%	14mg	2-year OS: 74% (95% CI: 60-88)	2-year PFS: 71% (95% CI: 56-87)	
Koehler et al.[18]	Germany	53	57	7.6 years	Median 20mg (range 8-24)	58%	n/a	34 months (95% CI 20.2-47.8)	12 months (95% CI 4.4-19.6)	
